# Mapping of Major *Fusarium* Head Blight Resistance from Canadian Wheat cv. AAC Tenacious

**DOI:** 10.3390/ijms21124497

**Published:** 2020-06-24

**Authors:** Raman Dhariwal, Maria A. Henriquez, Colin Hiebert, Curt A. McCartney, Harpinder S. Randhawa

**Affiliations:** 1 Agriculture and Agri-Food Canada, Lethbridge Research and Development Centre, Lethbridge, AB T1J 4B1, Canada; raman.dhariwal@canada.ca; 2 Agriculture and Agri-Food Canada, Morden Research and Development Centre, Morden, MB R6M 1Y5, Canada; mariaantonia.henriquez@canada.ca (M.A.H.); colin.hiebert@canada.ca (C.H.); curt.mccartney@canada.ca (C.A.M.)

**Keywords:** QTL mapping, doubled haploid, disease resistance, *Fusarium* head blight, plant height, days to anthesis, epistasis

## Abstract

*Fusarium* head blight (FHB) is one of the most devastating wheat disease due to its direct detrimental effects on grain-yield, quality and marketability. Resistant cultivars offer the most effective approach to manage FHB; however, the lack of different resistance resources is still a major bottleneck for wheat breeding programs. To identify and dissect FHB resistance, a doubled haploid wheat population produced from the Canadian spring wheat cvs AAC Innova and AAC Tenacious was phenotyped for FHB response variables incidence and severity, visual rating index (VRI), deoxynivalenol (DON) content, and agronomic traits days to anthesis (DTA) and plant height (PHT), followed by single nucleotide polymorphism (SNP) and simple sequence repeat (SSR) marker genotyping. A high-density map was constructed consisting of 10,328 markers, mapped on all 21 chromosomes with a map density of 0.35 cM/marker. Together, two major quantitative trait loci for FHB resistance were identified on chromosome 2D from AAC Tenacious; one of these loci on 2DS also colocated with loci for DTA and PHT. Another major locus for PHT, which cosegregates with locus for low DON, was also identified along with many minor and epistatic loci. QTL identified from AAC Tenacious may be useful to pyramid FHB resistance.

## 1. Introduction

*Fusarium* head blight (FHB), caused by *Fusarium graminearum* (Schwabe) (*Fg*) and another ~17 [1] species of the genus *Fusarium,* is one of the most devastating wheat diseases in the world due to its detrimental effects on grain-yield, quality and marketability. Moreover, contamination with *Fusarium* damaged kernels (FDK) and mycotoxins such as trichothecenes deoxynivalenol (DON), nivalenol and zearalenone [2] renders the grains unsuitable for food and feed. Recent FHB epidemics in the Canadian prairies resulted in significant economic impact on yield and grading losses to producers [3]. In western Canada alone, the eastern prairies have been most seriously affected with frequent outbreaks [3], but recently, concerns have arisen over the rapid spread of FHB in the western prairies [3]. FHB driven grade reductions caused significant economic losses in 2009, 2010 and 2012 (ranging from $2.9 to $8.7 million) in Alberta [4], especially in durum and highly susceptible soft white-spring wheat cultivars [3] but no serious outbreak was reported until 2016, when the downgrading of over 700,000 tons of wheat resulted in the loss of $12.8 million [5].

Although growing resistant cultivars has long been considered the most promising approach to alleviate the FHB associated risks [6], the development of FHB-resistant wheat cultivars (cvs) has been hindered due to a lack of knowledge, the complexities of resistance genetics and the absence of different resistance sources [7]. The complexities of the resistance genetics can be divided into two main categories: one which is characterized by different phenotypic responses or types of resistance within varietal resistance, and another which is associated with resistance but with unfavorable traits. Resistance to initial infection or disease incidence (DI, type I), the spread of symptoms within the head or disease severity (DS, type II) and DON accumulation (type III) are three well characterized phenotypic responses within complex varietal resistance, accompanied by three others: insensitivity to DON (type IV), resistance to FDK (type V) and tolerance or the ability to produce marketable grains in the presence of FHB (type VI) [7,8,9,10]. Associations between FHB resistance and a number of other traits such as days to anthesis (DTA) and plant height (PHT) [7,11,12,13,14,15,16] further complicate the genetics of FHB resistance which are controlled by polygenes/quantitative trait loci (QTL) that predominantly manifest in small additive effects and high genotype x environment interactions [17,18,19,20]. Venske et al. [21] identified as many as 65 meta-QTL for FHB resistance in wheat [21]. A few FHB-resistant QTL, such as *Fhb1* [22], *Fhb2* [23,24] and *Fhb5* [25], respectively on chromosome arms 3BS, 6BS, and 5AS, are the most-characterized and used QTL in breeding programs. These loci possess relatively large additive effects, and are sometimes treated as discrete loci. All three loci were found in FHB-resistant Chinese wheat cv Sumai 3 and some of its derivatives. *Fhb1* and *Fhb5* have also been fine mapped [26,27,28,29]. Another reported prominent resistance QTL is on chromosome arm 2DL, initially detected from a Chinese breeding line ‘Wuhan-1′ [16]. Moreover, three resistance loci, i.e., *Fhb3*, *Fhb6* and *Fhb7*, have been introgressed into wheat from its wild relatives *Leymus racemosus* [30], *Elymus tsukushiensis* [31] and *Thinopyrum ponticum* [32], respectively. A number of small-effect FHB resistance loci have also been detected from several north American and European wheat cvs and mapped to different chromosomes [19,33]. In addition to resistance QTL, a number of susceptibility factors have been identified from Sumai 3 [34,35,36] and Chinese Spring [37].

Recently, claims have been made that *Fhb1* has been cloned [38,39,40], and candidate genes from the QTL regions on wheat chromosome arms 2AS [41], 2DS [42] and 2DL [43,44] have been identified; however, there is still no consensus among the breeding community regarding the real genes. *PFT* (pore-forming toxin-like protein) [38] and *His* (histidine-rich calcium-binding protein) [39,40] genes, singly or as a haplotype of both [45], have been reported to be pivotal genes in the *Fhb1* region. Two recent studies [39,40] identified a critical deletion in the *His* gene as the causative mutation that gives rise to *Fhb1*-mediated resistance; however, these studies interpreted their findings differently. Su et al. [39] found that *His* is a susceptibility factor and the resistance is the result of a loss-of-function mutation, while Li et al. [40] observed that *His* is a resistance gene, and the same mutation results in a gain-of-function [46].

After decades of scientific research, our understanding about FHB resistance is still limited [6]. Although wheat cv Sumai 3 and its derived lines are seemingly the most studied so far, its host genetics are still a partial enigma. While efforts are still being made to understand *Fhb1*-mediated Sumai 3 resistance, there is increasing interest in understanding native FHB resistance in Canadian wheat cvs [47]. AAC Tenacious [48], an elite, highly FHB-resistant Canada Prairie Spring (CPS) wheat cv, is believed to carry its major resistance components from one of its progenitors, moderately resistant [49] Canadian heritage wheat cv Neepawa [50] (through male parent BW346) and FHB-resistant [48] female parent HY665. AAC Tenacious has excellent FHB resistance (likely better than Sumai 3). Despite its resistance response and parentage, it has not yet been explored for dissecting FHB resistance. The use of AAC Tenacious for genetic dissection of FHB resistance would be more beneficial for many breeding programs working on similar wheat classes/categories around the globe, since AAC Tenacious can be used directly for crossing due to its improved agronomic and quality traits compared to exotic resources and wild relatives of wheat. Therefore, a doubled haploid (DH) population developed from a cross of FHB susceptible Canada Western Special Purpose (CWSP) wheat cv AAC Innova [51] and AAC Tenacious was utilized to study FHB resistance. In this study, we identify major and stable FHB resistance from the Canadian wheat cultivar AAC Tenacious.

## 2. Results

### 2.1. Phenotypic Analysis: Varietal Resistance, Days to Anthesis and Plant Height

FHB nurseries have had good and homogeneous disease development over the years. The ANOVA showed significant differences among genotypes and environmental effect for DI, DS, visual rating index (VRI) and DON, and a genotype x environment effect for DON (Table 1). Among parents and common checks, while mean DI ranged from 2.5 to 9.1 in 2015, it was slightly higher in 2016 (ranged from 4.4 to 10.0), and very similar in 2017 (ranged from 2.2 to 10.0) (Supplementary Table S1). Similarly, DS was comparable over these years. Visual rating index (VRI) and DON response patterns were also quite comparable to DS over these years, except for a slightly higher index and DON accumulation of some checks and AAC Tenacious in 2016, an epidemic year (Supplementary Table S1). It seems that the higher index and DON in 2016 were largely due to the higher DI in 2016, as DS was similar over these years. Where both parents showed different phenotypes for DI, DS, VRI and DON in different years, AAC Tenacious showed the lowest DI, DS, VRI and DON among parents and checks. Mean DI, DS and VRI data showed that Sadash and CDC Teal were the most susceptible cvs among checks (Supplementary Table S1). DON data showed that while Sadash was one of two most susceptible cvs, AC Morse had highest DON accumulation. CDC Teal, AAC Indus and AAC Foray had similar DON accumulation (Supplementary Table S1). 

**Figure 1:**
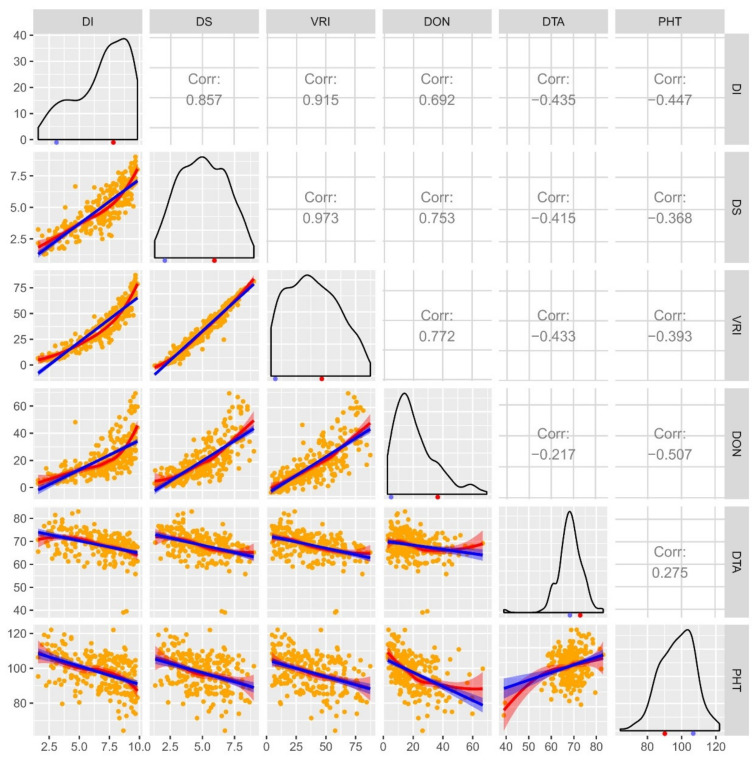
A combined correlogram of doubled haploid (DH) population of cross AAC Innova x AAC Tenacious drawn using pooled phenotypic data (average of all environments) of four *Fusarium* head blight (FHB) traits disease incidence (DI; measured on a scale of 0-10), severity (DS; measured on a scale of 0-10), visual rating index (VRI; DI x DS) and deoxynivalenol (DON; measured in ppm) content, and days to anthesis (DTA; days) and plant height (PHT; measured in cm) evaluated at Lethbridge and/or Morden in Canada. Scatterplots with regression lines, linear (blue) and exponential (red), for each pair of traits are drawn on the lower-left part of the plot matrix. Orange dots in scatterplots represent DH lines. Pearson correlations are displayed on the upper-right part of the plot matrix. Frequency distribution plots for each trait are shown on the diagonal. The means of the parental genotypes AAC Innova and AAC Tenacious are indicated by red and blue dots, respectively, beneath frequency distribution plots.

Where the population frequency distribution of DI was skewed towards susceptibility, it was skewed towards resistance for DON. Distribution of DS and VRI were close to normal (Figure 1). Over the years, while population means of DI, DS, VRI and DON were within the range of the two parents (Supplementary Table S1), both upward and downward transgressive segregants were observed (Figure 1). Downward transgressive segregants indicate the presence of superior genes/QTL for FHB resistance in both parents [52].

ANOVA also showed significant differences among genotypes and environmental effect for both agronomic traits, i.e., DTA and PHT (Supplementary Tables S2 and S3), in addition to a genotype x environment interaction effect for DTA (Supplementary Table S2). Though both parents flower relatively late in the growing season, AAC Tenacious had anthesis 3.3 days earlier than AAC Innova (mean flowering in 70.1 days) (Supplementary Table S4). The population frequency distribution for DTA was near normal with a mean anthesis date within the range of two parents, but many upward and downward transgressive segregants were observed (Figure 1). AAC Innova had more desirable PHT (89.2 cm; 16.1 cm less) than AAC Tenacious (Supplementary Table S4). The DH population differed broadly for PHT, with the shortest and tallest DHs deviating by 56.7 cm where the mean of population was 98.0 cm. Similar to DTA, distribution was near normal for PHT, and both upward and downward transgressive segregants were observed (Figure 1).

A correlation analysis demonstrated positive relationships among DI, DS, VRI and DON, and between DTA and PHT, but a negative relationship between FHB related traits/variables and agronomic traits (DTA and PHT; Figure 1). Regression line analysis showed moderate to high contributions of DI and DS to DON accumulation (Figure 1), indicating the usefulness of DI and DS data for determining FHB resistance and DON accumulation. 

### 2.2. Genetic Map

A total of 10,328 high quality polymorphic single nucleotide polymorphism (SNP) markers from the wheat 90K SNP Infinium iSelect assay and simple sequence repeat (SSR) molecular markers were genetically mapped to 25 linkage groups (LGs) belonging to 21 wheat chromosomes in the AAC Innova x AAC Tenacious population (Table 2 and Supplementary Table S5). This map spanned a total of 3613.36 cM map distance (on average 172.06 cM per chromosome) with an average density of one marker every 0.35 cM (or 2.34 markers per cM) (Table 2). The highest number of markers were mapped to wheat homoeologous group 1 chromosomes (2565 markers; density = 6.29 markers/cM) and the lowest to group 4 chromosomes (760 markers; density = 1.72 markers/cM) (Table 2). On the other hand, where genome B showed the highest marker coverage (5077 markers; density = 3.96 markers/cM), i.e., slightly higher than the genome A (4112 markers; density = 2.75 markers/cM), genome D showed the lowest coverage (1139 markers; density = 1.36 markers/cM) (Table 2). Despite the mapping of vast number of markers and their high densities, the number of linkage bins spanned on all 21 chromosomes was 1543. This led to an average density of one linkage bin every 2.34 cM (ranging from 0.53 to 42.70) (Table 2; Supplementary Table S5). This showed that the distribution of loci along the chromosomal arms was uneven, and clusters were formed at certain regions; however, only 65 linkage bins exceeded 10.00 cM. A total of 438 and 263 (out of total 10,328) markers were newly mapped which were not present on any previously published individual wheat consensus maps [53,54,55,56,57] or integrated wheat consensus map [57], respectively. The order of the markers in the present study was largely in agreement (Supplementary Table S5) with previously published consensus maps [53,54,55,56,57].

### 2.3. QTL Analysis

#### 2.3.1. FHB Resistance and DON Content

**Figure 2:**
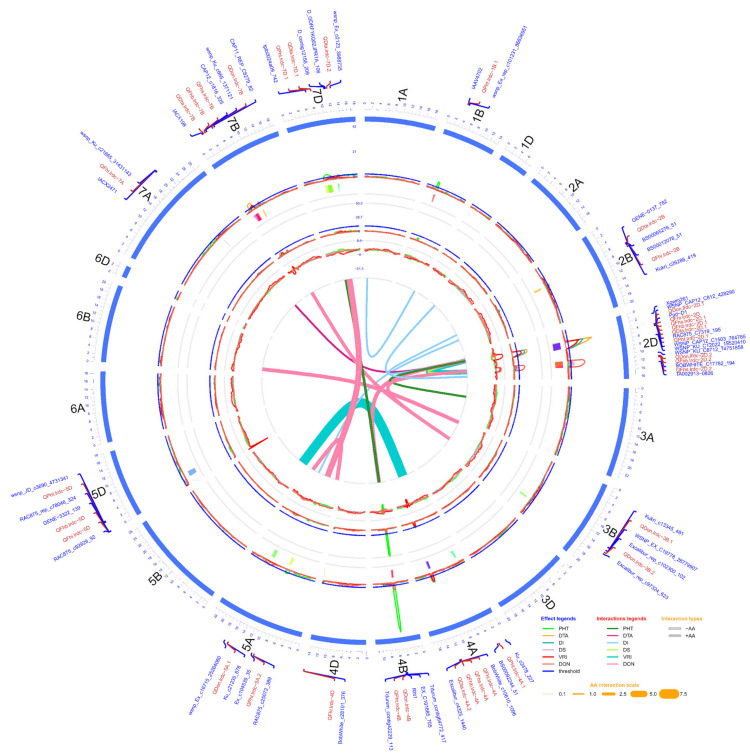
Circos diagram of linkage and QTL map developed using pooled phenotypic data (average of all environments) of doubled haploid population produced from the cross AAC Innova x AAC Tenacious. The outermost circle/track shows the 21 chromosomes (1A -7D) arranged in a clockwise direction with selected QTL-linked/flanking markers in 0.1X scale (cM). Three inner circles and line connections in the middle represent the mean LOD score (second track from outside), % phenotypic variation (*R*^2^) explained (third track from outside), additive effect (AE) (fourth track from outside) of individual QTL and epistatic effect (AA) of digenic QTL x QTL interactions (line connections in the middle) for different measured traits. In the second and third tracks from outside, blue lines show a LOD threshold of 2.5 and *R*^2^ threshold of 10%, respectively. QTL confidence intervals are shown in different colors beneath the QTL scans in second track from outside. LOD, *R*^2^ and AE peaks of different traits are represented in different colors as shown in the effect legends in the lower-right corner of the diagram. Three most significant and stable QTL identified were on chromosomes 2D and 4B. AA interactions between QTL pairs of the same or different linkage groups for each trait are represented in middle of diagram by line connections of different colors, as shown in the interaction legend in the lower-right corner of the diagram. Negative and positive AA interactions are represented by hollow and solid lines, respectively. The width of the line connections represent the strength of AA effect, as shown in the AA interaction scale in the lower-right corner of diagram.

QTL analyses were carried out separately for DI, DS, VRI and DON using phenotypic data of individual environments, as well as pooled (average of all environments) data. A total of seven main effect QTL for resistance to DI (type-I resistance), eleven to DS (type-II resistance), nine to index (VRI), and nine to DON (type-III resistance) were identified in one or more environments (Figure 2; Table 3 and Supplementary Table S6). Twenty of these QTL, along with two additional suggestive [58] QTL, one for DI on chromosome 7A and another for DON on chromosome 3B, were identified using pooled data (Table 3). While the phenotypic variation (*R*^2^) explained by these 22 QTL ranged from 2.10 (*QFhi.lrdc-7A*) to 34.50% (*QDon.lrdc-2D.2*) (Table 3), only 10 of these explained >10% *R*^2^ using pooled data, irrespective of their individual environment values. Notably, only one stable QTL for DI (*QFhi.lrdc-2D*) on chromosome 2DS, two for DS (*QFhs.lrdc-2D.1* and *QFhs.lrdc-4A*) on chromosomes 2DS and 4A, two for VRI (*QFhb.lrdc-2D.1* and *QFhb.lrdc-4A*) on chromosomes 2DS and 4A, and one for DON (*QDon.lrdc-2D.2*) on chromosome arm 2DL were detected across the tested environments (Supplementary Table S6); however, only those present on chromosome 2D were considered major QTL based on phenotypic variation explained.

In addition to the aforementioned main effect QTL, 13 digenic epistasis interactions of 20 different loci were also identified (Figure 2; Table 4). All these epistasis interactions possessed a significant (*p* ≤ 0.05) additive x additive effect (Table 4). Notably, based on within 10.0 cM criteria, seven loci or chromosomal regions which appeared in digenic epistasis interactions were also identified during the main effect QTL analysis. These included QTL regions close to 72.6-82.5 cM on chromosome (arm) 2B (overlapping interval of *QFhs.lrdc-2B* and *QFhb.lrdc-2B*), 28.5-48.4 cM on 2DS (overlapping interval of *QFhi.lrdc-2D*, *QFhs.lrdc-2D.1* and *QFhb.lrdc-2D.1*), 106.2-120.4 cM on 2DL (overlapping interval of *QDon.lrdc-2D.2*), 130.1-135.3 cM on 3B (overlapping interval of *QDon.lrdc-3B.1*), 47.7-50.0 cM on 4B (overlapping interval of *QDon.lrdc-4B*), 184.7-196.9 cM on 5A (overlapping interval of *QDon.lrdc-5A.2*) and 52.1-58.3 cM on 7BS (overlapping interval of *QDon.lrdc-7B*) (Table 3 and Table 4 and Supplementary Table S6). Out of all 20 epistasis loci, three QTL regions on chromosomes (or arms) 2DS, 2DL and 3B, as designated above with the respective QTL, were detected in interactions with more than one gene/QTL, either for the same or different response variable/trait (Table 4).

#### 2.3.2. Days to Anthesis and Plant Height

Similar to FHB response, QTL analyses were carried out separately for DTA and PHT using phenotypic data of individual environments as well as pooled data. A total of ten main effect QTL for DTA and fourteen for PHT were identified in one or more environments using data of individual environments (Figure 2; Table 3 and Supplementary Table S6). Twelve of these QTL along with one additional suggestive [58] QTL for PHT on chromosome 5D were identified using pooled data (Table 3). While these 13 QTL explained 2.00 (*QPht.lrdc-4A.1*) to 53.00% (*QPht.lrdc-4B*) *R*^2^ using pooled data (Table 3), only one DTA QTL (*QDta.lrdc-2D.1*) and two PHT QTL (*QPht.lrdc-2D.1* and *QPht.lrdc-4B*) explained ≥10% of the *R*^2^ and were considered major QTLs. However, only two stable DTA QTL (*QDta.lrdc-2D.1* and *QDta.lrdc-7B*) and one PHT QTL (*QPht.lrdc-4B*) were detected across the tested environments (Supplementary Table S6). Shorter DTA and reduced PHT alleles for different QTL were attributable to both parents (Table 3 and Supplementary Table S6).

In addition to the main effect QTL for DTA and PHT, three digenic epistasis interactions of six different loci were also identified (Figure 2; Table 4). All epistasis QTL also showed significant additive x additive effects (Table 4). Unlike FHB related QTL, the majority of epistasis loci (except one QTL close to 234.8 cM on 7A) overlapped with QTL regions identified during the main effect QTL analysis.

#### 2.3.3. Pleiotropic QTL 

Main effect QTL positions of FHB response variables/traits DI, DS, VRI and DON, and agronomic traits were compared. Chromosomal intervals which harboured two or more QTL within 10.0 cM were considered shared or common QTL regions, while the rest of the QTL intervals (>10.0 cM apart) were considered unique QTL regions. This analysis identified a total of 25 unique (10 for FHB variables or related traits on chromosomes 1A, 2A, 3B, 4B, 5A, 6A, 7A and 7B; 4 for DTA on 2D, 4A, 5B and 7D; and 11 for PHT on 1A, 1B, 2D, 3A, 4A, 5A, 5D, and 7D) and 11 shared QTL regions on different chromosomes identified from the QTL results that utilized phenotypic data belonging to all individual environments and pooled data (Supplementary Table S7). Notably, while all 11 shared regions harbouring QTL for FHB related variable(s)/traits, seven of these also harboured QTL for one of two agronomic traits, i.e., DTA (on 2B, 5A and 7B) and PHT (on 4B), or both (on 2DS, 4A and 7D). These seven were considered pleiotropic loci. On the other hand, only four shared regions harboured QTL solitarily for FHB related variables/traits, and were located on chromosomes 2DL, 3B, 4D and 5D (Supplementary Table S7). Only the 2DL region of these had a major effect QTL. Where all these regions harbouring FHB index QTL, 4D and 5D regions provided both type-I and -II resistance, the 2DL region provided both type-II and -III resistance and the 3B region provided only type-II resistance. Reduced FHB alleles at different loci in three (2DL, 3B and 5D) out of four regions were derived from AAC Tenacious. Among seven pleiotropic QTL regions, while one on chromosome arm 2DS harboured QTL for type-I, -II and -III resistances along with FHB index, the remaining six harboured QTL for ≤2 type of resistance with or without index QTL (Supplementary Table S7). Reduced FHB alleles at different loci in the 2B, 2DS, 4B and 5A regions were derived from AAC Tenacious, while in three other regions, i.e., 4A, 7BS and 7D, they were derived from AAC Innova (Supplementary Table S7). 

**Figure 3:**
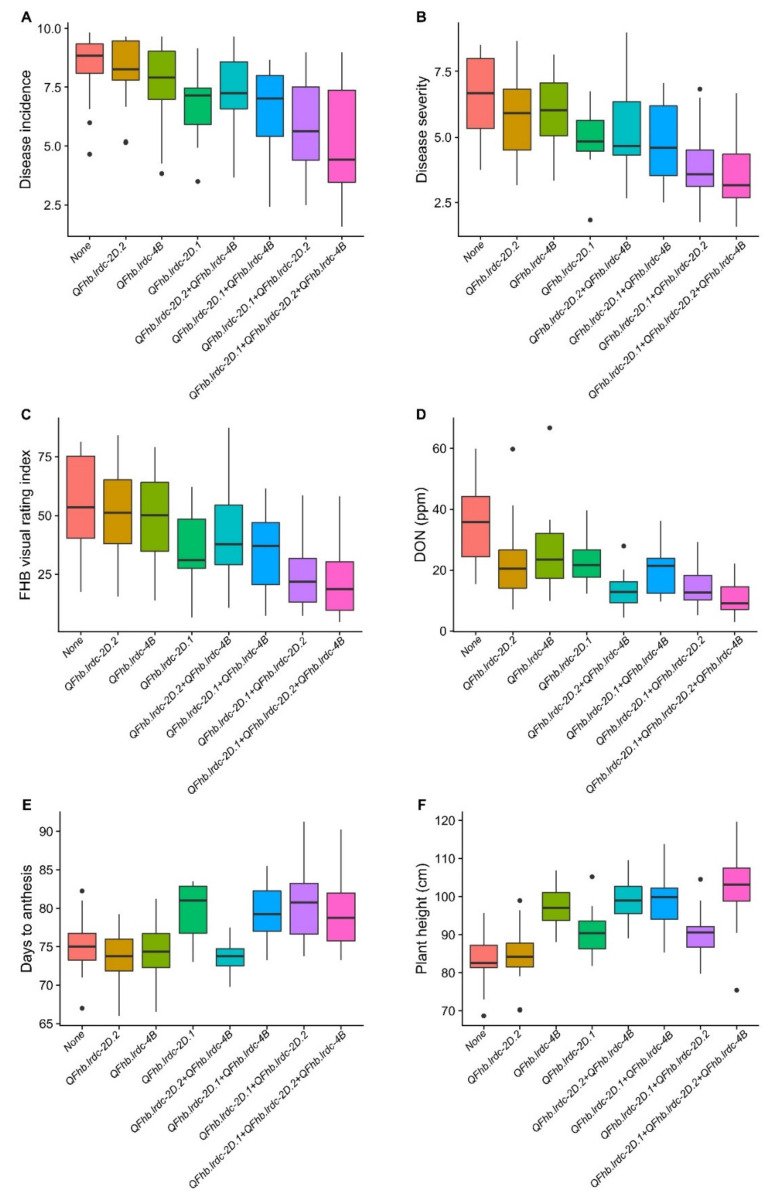
Boxplot distributions of doubled haploid (DH) population produced from the cross AAC Innova x AAC Tenacious. Effects of positive alleles of single QTL (*QFhb.lrdc-2D.1*, *QFhb.lrdc-2D.2* and *QFhb.lrdc-4B*) and their combinations on average *Fusarium* head blight incidence (**A**), severity (**B**), visual rating index (**C**), deoxynivalenol (DON) accumulation (**D**), days to anthesis (**E**) and plant height (**F**) are depicted alongside negative alleles at all three loci using pooled phenotypic data (average of all environments). Quartiles and medians are represented by boxes and continuous lines, respectively. Whiskers extend to the farthest points that are not outliers, whilst outliers are shown as black dots.

Three regions (solitarily FHB responsive 2DL and pleiotropic 2DS and 4B) which had QTL, explaining the largest phenotypic variation, and all derived from AAC Tenacious were assessed individually and in combination for their effect on different traits in DH population (Figure 3). It was observed that while individually, 2DS region contributed maximum to lower DI, DS and VRI, the 2DL region contributed towards a maximum reduction of DON content. Though the 4B region reduced DI, DS, VRI and DON, its effect was smaller on these response variables/traits compared to the other two regions (Figure 3). Similar results were observed in combinations (Figure 3). Interestingly, where low FHB 2DL and 4B alleles had no significant effect on days to anthesis, 2DS low FHB allele delayed anthesis time significantly (Figure 3). Conversely, except for the low FHB 2DL allele, both 2DS and 4B low FHB alleles increased plant height, with the effect of 4B being very prominent (Figure 3). 

## 3. Discussion

There is increasing interest in identifying and characterizing native FHB resistance, as breeders have struggled to achieve sufficient progress in improving FHB resistance using exotic resources [47]. Although some attempts [15,47,59,60] have been made recently to genetically determine native FHB resistance in Europe and north America, there is an ever-increasing need to identify new resources, as FHB is spreading to previously unaffected areas [3]. In the present study, a spring wheat DH population, its parents and check cvs were subjected to extensive phenotyping for FHB-DI, -DS, -VRI and DON, as well as agronomic traits DTA and PHT, followed by high-throughput genotyping. The largely different phenotypic expression of both Canadian parents, i.e., AAC Innova, being susceptible, and AAC Tenacious, being highly resistant (Supplementary Table S1), and the broad genetic variation (Table 1) make this population ideal for dissecting FHB resistance. This study is the first effort to unravel the FHB resistance of AAC Tenacious, i.e., the first FHB-resistant wheat cultivar in Canada, which is supposed to carry a native or Canadian climate-adapted resistance component from its FHB-resistant female parent HY665 [48] and moderately FHB-resistant Canadian wheat cv Neepawa [49], a grandparent in lineage of its male parent BW346.

We utilized the Infinium iSelect 90K SNP assay [54] and a few other markers for genotyping, which resulted in the mapping of 10,328 markers representing 1543 linkage bins on all 21 wheat chromosomes. In the present study, the marker density of 2.34 markers per cM was comparable to the recently published individual wheat maps of two populations, ‘Doumai × Shi 4185′ and ‘Gaocheng 8901 × Zhoumai 16′, where marker densities were 3.85 and 3.0 markers/cM, respectively [57]. Conversely, the marker coverage of the present map is also consistent with previous studies, where B-genome had the highest marker coverage, followed by the A and D genomes [53,54,57,61,62]. Additionally, we identified at least 263 new markers that were not present on any individual or integrated previously published wheat consensus map [53,54,55,57]. These SNPs could be due to the use of the native germplasm, which was not present in previous studies [52] and helped us to explore the native FHB resistance in this population.

A total of 21 FHB responsive loci or chromosomal regions (15 from AAC Tenacious and 6 from AAC Innova), which involved both unique loci (10) and shared QTL regions (11; 4 solitarily FHB related and 7 most likely pleiotropic), were identified by this study (Supplementary Table S7). Notably, unique QTL, which were not detected in all environments, explained <10.0 % *R*^2^, and were thus considered small effect [63] suggestive [58] QTL. Instead, three shared QTL regions (i.e., 2DS, 2DL and 4B) harbored some major QTL (explained >10.0 % *R*^2^) for FHB response variables/traits which also showed high LOD score (>5.0) and additive effect, and were also detected over all tested environments and pooled data (Supplementary Table S7). These major and stable QTL in these shared regions include FHB-resistant QTL *QFhi.lrdc-2D*, *QFhs.lrdc-2D.1*, *QFhb.lrdc-2D.1* and *QDon.lrdc-2D.2*, and DTA and PHT QTL *QDta.lrdc-2D.1*, *QPht.lrdc-2D.1*, and *QPht.lrdc-4B*. Broadly, the 2DL region of the three shared regions was the only specific form of FHB resistance; the rest appeared to result from the pleotropic effect of the loci also affecting either both DTA and PHT (2DS) or just PHT (4B). Interestingly, AAC Tenacious contributed to the resistance alleles at all major and stable loci in these regions. These QTL regions are discussed in greater detail below.

The 2DS FHB-resistant QTL had highest peaks, ranging from 40.6–40.9 cM, on the chromosome arm 2DS, which colocated with the photoperiod responsive gene *Ppd-D1* (40.76 cM). The DTA QTL *QDta.lrdc-2D.1* and PHT QTL *QPht.lrdc-2D.1* confidence interval also overlapped 2DS FHB resistance-related QTL (40.6–40.9 cM) and the *Ppd-D1* gene (40.76 cM). Associations among FHB, DTA and PHT are very common and have been thoroughly studied in wheat. FHB-resistant QTL have been shown to have overlapping anthesis time and plant height QTL with the European winter wheat population Renan/Recital [15].

Resistance loci on the chromosome arm 2DS were also identified in previous studies on wheat genotypes Alondra [64], Biscay [20], C615 [65], DH181 [66], Ernie [60], Furore [67], Gamenya [42,68], Jagger [69], Kenyon [47], Maringa [16], Massey [60], Naxos [11,70], Romanus [20], Shanghai-3 [11], Wangshuibai [71,72], Y1193-6 [36] and Yanzhan 1 [73]. The majority of previously published 2DS resistance loci mapped close to/overlap either one of/or two important markers loci, reduced height gene *Rht8* [74] linked marker locus *Xgwm261* in genotypes Furore, Gamenya, Kenyon, Maringa, Massey, Romanus, Wangshuibai, Y1193-6 and Yanzhan 1 [16,20,36,42,47,60,67,68,71,72,73] and photoperiod responsive gene *Ppd-D1* in cvs/genotypes Ernie, Kenyon, Massey and Naxos [47,60,70]. Previously, it has been speculated that both the tall allele of *Rht8* and the photoperiod-sensitive allele of *Ppd-D1* may be causal genes for two different 2DS FHB-resistant QTL in cv Kenyon [47]. In another study, Liu et al. [60] reported that the photoperiod-sensitive allele *Ppd-D1b* pleiotropically reduced FHB susceptibility and increased plant height in Becker/Massey and Ernie/MO 94-317 populations. It was observed that previously mapped 2DS QTL from cvs/genotypes Alondra, Ernie, Furore, Gamenya, Jagger, Kenyon, Massey, Shanghai-3, Wangshuibai and Y1193-6 [11,36,47,60,64,67,68,69,71] not only overlap with the *Ppd-D1* region, but also with each other when compared to the wheat reference genome using their linked marker sequences [75]. Since DTA and PHT loci, i.e., *QDta.lrdc-2D.1* and *QPht.lrdc-2D.1*, were also identified in this 2DS region in present study, it became important to know whether the phenotypic effects of this interval were due to *Rht8* or *Ppd-D1*. Thus, we genotyped the AAC Innova x AAC Tenacious population with *Ppd-D1* and *Xgwm261* markers. We observed that while AAC Innova carried a photoperiod-insensitive allele, i.e., *Ppd-D1a*, and a reduced brassinosteroid-sensitivity semidwarf allele, i.e., *Rht8c*, at *Xgwm261* locus [76], AAC Tenacious carried the photoperiod-sensitive allele *Ppd-D1b* and the brassinosteroid-sensitive tall allele *Rht8a* [76]. These results seem obvious, as AAC Innova is a semidwarf cv, while AAC Tenacious is a tall cv, and do not reject the potential role of *Rht8* affecting FHB response. However, the linkage and QTL mapping conducted using these markers eliminated the role of *Rht8* in 2DS-mediated phenotypic effects. Our results showed the highest peak of all 2DS QTL (*QFhi.lrdc-2D*, *QFhs.lrdc-2D.1*, *QFhb.lrdc-2D.1*, *QDon.lrdc-2D.1*, *QDta.lrdc-2D.1* and *QPht.lrdc-2D.1*) mapped close to/overlap *Ppd-D1*, while the *Rht8* marker locus *Xgwm261* was mapped at 27.2 and 24.4 cM distal to *Ppd-D1* and 2DS QTL interval flanking marker wsnp_CAP12_c812_428290, respectively. These observations also indicate that AAC Innova and AAC Tenacious may be monomorphic at the *Rht8* locus, despite the polymorphism at *Xgwm261*, which is perhaps due to historic recombination events between the two loci. Thus, the AAC Tenacious derived-photoperiod sensitive allele *Ppd-D1b* seems to be responsible for the phenotypic expression of 2DS QTL, particularly of *QDta.lrdc-2D.1* and *QPht.lrdc-2D.1*, as *Ppd-D1b* is known to strongly regulate anthesis time [77], and has also been previously reported to pleiotropically control the height by shortening the plant life cycle [78]. Moreover, though all FHB, DTA and PHT QTL spanned the same interval, the highest peaks of disease-responsive (*QFhi.lrdc-2D*, *QFhs.lrdc-2D.1*, *QFhb.lrdc-2D.1* and *QDon.lrdc-2D.1*) and agronomic (*QDta.lrdc-2D.1* and *QPht.lrdc-2D.1*) QTL were separated by ~1.0 cM (Table 3), which is not statistically significant. Thus, the FHB resistance in this region may be due to either (i) *Ppd-D1b*, or (ii) a gene other than *Ppd-D1*, but with a linkage drag between the *Ppd-D1b-*sensitive allele and FHB resistance [1]. Further experiments involving a large fine mapping population are required to confirm the presence of two different loci in this region.

The second most important locus/chromosomal region identified during the present study was the solitarily FHB resistance-specific 2DL region. It harbored three important QTL, i.e., *QFhs.lrdc-2D.2* (115.6 cM), *QFhb.lrdc-2D.2* (114.6 cM) and *QDon.lrdc-2D.2* (112.6 cM). While all of these QTL explained >10% phenotypic variation and are major loci, *QDon.lrdc-2D.2* explained up to 34.5% phenotypic variation for DON, was stable over all of the tested environments and had no linkage drag. Interestingly, AAC Tenacious contributed resistance alleles at all these QTL in this region, and thus, all these QTL could be the result of a single gene. QTL on chromosome arm 2DL have been identified in previous individual QTL mapping studies utilizing cvs/lines of different origin, i.e., Canada (cv Kenyon), China (cvs Changjiang 9306 or CJ 9306, CS-SM3-7ADS, Shanghai-3, Wangshuibai, Wuhan-1), CIMMYT, Mexico (cvs C615, Soru#1 and SYN1), Swiss (cv Arina), and USA (cvs VA00W-38 and CASS94), [11,16,47,59,65,70,79,80,81,82,83,84,85]. Moreover, it appears that the 2DL QTL region from the present study overlapped with the 2DL QTL regions of at least Arina [83], CASS94 [82], CJ 9306 [79], CS-SM3-7ADS [85], Kenyon [47], Shanghai-3 [11], Soru#1 [70], SYN1 [80], Wangshuibai [81,84] and Wuhan-1 [16] when compared with the wheat reference genome using their linked marker sequences [75]. Interestingly, the 2DL QTL are reported to explain >10% (up to 60%) total phenotypic variation in the majority of the previous studies [47,70,79,80,81,82,84] with the exception of a few, such as QTL derived from Arina [83], Shanghai-3 [11] and Wuhan-1 [16]. This showed that QTL on the chromosome arm 2DL were not only detected repeatedly during different studies, but also that their expression was high, and they expressed in different genetic backgrounds, as witnessed by their widespread presence in global FHB-resistant wheat germplasm. With these qualities, in particular the absence of linkage drags or pleiotropic effect, and being a major contributor of DON reduction, it is considered a highly promising QTL for developing FHB-resistant cultivars.

Another important region identified during this study was on chromosome 4B. It harbored FHB-responsive QTL *QFhi.lrdc-4B*, *QFhb.lrdc-4B* and *QDon.lrdc-4B*, and PHT QTL *QPht.lrdc-4B*. Though *QDon.lrdc-4B* was also a major QTL in this region, *QPht.lrdc-4B* was the only stable QTL detected in all tested environments. *QPht.lrdc-4B* mapped to the same genomic interval as of *Rht1* (*Rht-B1*) on chromosome 4B during synteny and collinearity analysis using *QPht.lrdc-4B* flanking marker sequences (*Ex_c101685_705* and *Tdurum_contig42229_113*) and *Rht-B1a* gene sequence (GenBank Accession: FR719732) utilizing IWGSC RefSeq v2.0. AAC Tenacious contributed both increased height and DON reduction alleles along with DI and VRI reduction at this region. We found that AAC Tenacious possesses a gibberellic acid (GA)-sensitive, tall height allele of *Rht1*. Earlier studies reported an association between *Rht-B1a* (GA-sensitive allele) and low FHB as well as tall height [11,86]. A recent report showed decreased FHB spread in GA treated wheat plants [87]. Interestingly, despite the association of tall height with FHB resistance, we found five highly FHB-resistant DH lines carrying a GA-sensitive allele but with semidwarf statures in comparison to several DH lines carrying an insensitive allele, perhaps due to transgressive segregation at some other *Rht* loci. *Rht24* is one such gene which exhibits reduced plant height without influencing FHB [88]. In addition to the direct effect of *QPht.lrdc-4B*, it also reduced DON in epistasis with *Ppd-D1* during this study. Guo et al. [89] observed that the *Ppd-D1b* and *Rht-D1a* alleles affected the different floral developmental stages, extending the duration of stem elongation phase, floret primordium initiation and booting. It appears that sensitive alleles at the *Ppd-D1* and *Rht* loci coordinate for different developmental activities and FHB resistance. Although the favorable alleles for DI, VRI and DON at this region were from AAC Tenacious, even in some semidwarf DHs with a GA-sensitive allele, its use in breeding may still result in a large trade-off, as the GA-sensitive allele of *Rht1* is significantly associated with increased PHT. Thus, it would be more beneficial to deploy other resistance QTL to increase the resistance of genotypes carrying either *Rht1* or other genes such as *Rht24*.

In addition to the aforementioned QTL, type-II and index QTL *QFhs.lrdc-4A* and *QFhb.lrdc-4A*, DTA QTL *QDta.lrdc-7B* and PHT QTL *QPht.lrdc-7D.1*, that belong to three shared regions on chromosomes 4A, 7B and 7D, were also detected in all environments and utilizing pooled data; however, these QTL generally explained <10.0% *R*^2^ in individual environments. In these regions, favourable alleles for FHB resistance-related QTL were contributed to by AAC Innova, while AAC Tenacious contributed favourable alleles for DTA and PHT. Based on comparative mapping, it seems that *QFhs.lrdc-4A* and *QFhb.lrdc-4A* mapped to the similar location as 4A QTL in 86ISMN 2137 [47], and *QPht.lrdc-7D.1* to a similar location as 7D FHB-resistant QTL from Kenyon, Wesley-*Fhb1*-BC56 and AL-107-6106 [47,90].

The phenotypic effect of a gene or QTL allele at a locus may vary in different genetic backgrounds because of epistatic interaction with other gene loci. This means that an apparently “favorable” QTL allele at a locus may show a “neutral” or “unfavorable” phenotype effect in a new genetic background [91]. An understanding of epistatic loci is vital for breeding wheat, since epistasis not only complicates the genotype–phenotype relationship, but also affects the rate of genetic gain [91]. In this study, a number of epistasis loci involved in regulating FHB expression and other traits have been identified. Interestingly, seven FHB-influencing and five agronomic traits loci, involved in digenic epistatic interactions, were also identified during the main effect QTL analysis, clearly suggesting the influence of epistasis on the additive effect of the main effect QTL. Despite their importance and possible roles in crop improvement, epistatic loci influencing FHB are seldom reported, except in a few studies on wheat [47,85] and triticale [52]. This could be due to many factors, including the complexity of FHB phenotypic response and the less attractive minor effects resulting from individual interactions.

In summary, the results of the present study add to our knowledge of the unique, pleotropic and epistatic FHB-resistance QTL derived from Canadian wheat cvs AAC Innova and AAC Tenacious. More particularly, the three major FHB-resistance QTL identified during present study were derived from AAC Tenacious. These QTL will not only be valuable for Canadian wheat breeders, but may be useful for breeders in other growing regions. These QTL, along with the resources generated in this study, including the high-density SNP map and the DH lines with different gene/QTL combinations, will facilitate the development of FHB-resistant cvs using marker-assisted breeding, and will also help in map-based cloning.

## 4. Materials and Methods

### 4.1. Plant Material

A doubled haploid mapping population (224 lines) was generated from a biparental cross AAC Innova x AAC Tenacious using the wheat-maize pollination technique [92] at the Lethbridge Research and Development Centre, Lethbridge, AB to genetically examine FHB resistance and associated traits. AAC Innova is an FHB-susceptible, semidwarf cultivar, while AAC Tenacious is a resistant, tall cultivar. The cv AAC Innova was developed from the cross AC Andrew/N9195 (L01164) [51]. The resistant cv AAC Tenacious was derived from the cross HY665/BW346 [48]. The female parent, HY665, which was supposed to be a FHB-resistant source, was derived from the cross 96W137//Howell/HY617BSR [48]. The male parent, BW346, is believed to carry FHB resistance from Canadian wheat cv Neepawa, and had the pedigree RL4802//(96MHN5295-1)BW174*2/Clark [48]. 

A number of Canadian wheat cvs/breeding lines developed and maintained at Agriculture and Agri-Food Canada with different levels of resistance were also used as checks for comparisons in nurseries; these included the FHB-resistant breeding line FHB 37, the moderately resistant cv AAC Penhold, the intermediate cv AC Cora, the moderately susceptible cvs AAC Foray, Carberry, AAC Indus and 5602 HR and the susceptible cvs AC Morse, CDC Teal and Sadash.

### 4.2. Fusarium Head Blight (FHB) Phenotyping and Deoxynivalenol (DON) Assessment

The doubled haploid lines, their parents and checks were screened in replicated trials in the FHB nurseries at Morden, MB, Canada in 2015, 2016 and 2017. Trial entries and checks were replicated in a randomized block design. The entries were planted in a single 1-m-long row using a six-row cassette Row XL, Wintersteiger planter. The rows were spaced 30 cm apart with passes of 0.5 m between each set of six rows. *Fg* corn kernel inoculum was prepared using four *Fg* isolates HSW-15-39 (3-ADON), HSW-15-87 (3-ADON), HSW-15-27 (15-ADON) and HSW-15-57 (15-ADON). The inoculum was dispersed at a rate of 8 g per row on biweekly intervals, starting when earliest lines reached a 4–5 leaf stage. The application of the inoculum was followed by irrigation three times a week using Cadman Irrigations Travellers with Briggs booms. Visual observations were taken at 18 to 21 days postinoculation for infected heads (disease incidence; DI) and spikelets (disease severity; DS) on a scale of 0 to 10, and used to calculate the FHB visual rating index (VRI: DI × DS) [93]. Matured wheat heads were harvested from the center of each plot, dried and threshed using an Almaco (Nevada, IA) stationary thresher without the use of a fan, followed by cleaning the seed samples by hand to prevent the loss of *Fusarium*-damaged kernels. One 10 g aliquot from the two replications of each line/cultivar were ground using a Romermill (Model 2A, Romer Labs Inc., Union, MO, USA) to make a whole-grain flour. Quantification of DON was carried out using two 1-g aliquots sampled from each ground sample using EZ-Quant^®^ Vomitoxin ELISA kit (Diagnostix, Ltd., Mississauga, ON, Canada) with an accuracy of 0.5 ppm [94].

### 4.3. Days to Anthesis and Plant Height Phenotyping

Days to anthesis (DTA; Z00-61) was measured as the number of days from planting to Zadoks growth stage 61, when a few anthers started to emerge at the middle of spikes [95]. DTA was recorded in green house experiments in 2017, field experiments in 2018 and 2019 at Lethbridge, AB and field experiments in 2017 at Morden, MB.

Plant height (PHT; cm) was measured in green house experiments in 2017, field experiments in 2017, 2018 and 2019 at Lethbridge, AB and field experiments at Morden, MB in 2017. All measurements were taken on at least three plants in green house conditions and five plants per line/plot in field conditions.

### 4.4. Phenotypic Data Evaluation

The phenotypic data was subjected to ANOVA using the agricolae (version 1.2–4) package of the R (R version 3.2.3) software [96]. For the ANOVA model, DHs, their parents and checks were considered fixed effects, while environments and blocks were considered random effects. ANOVA was conducted both within and across environments. A combined ANOVA table for all FHB related traits was generated. Pearson correlations and regression between traits and scatterplots were calculated using the R package GGally software [97].

### 4.5. Genotyping and Linkage and Quantitative Trait Loci (QTL) Mapping

A total of 188 DH lines and their parents (AAC Innova and AAC Tenacious) were used for genotyping. Two seeds from each line were seeded in 2 x 96 cell seed planting trays (parents repeated over both trays) in a soil mixture of Turface (9.07 kg), Peat Moss (0.907 kg) and Vermiculite (0.06 m^3^). Leaf tissue samples were collected from 10 days old seedlings followed by isolation of DNA using DNeasy 96 Plant Kit (Qiagen Inc., Valencia, CA, USA) following Dhariwal et al. [52]. Quant-iT™ PicoGreen^®^ dsDNA Assay Kit (Thermo Fisher Scientific Inc., Bartlesville, OK, USA) was used to quantify DNA samples, followed by dilutions of DNA samples to 50 ng/µl. Marker genotyping was carried out using wheat 90K Infinium iSelect SNP assay, functional marker *Ppd-D1*, and simple sequence repeat (SSR) marker *Xgwm261*. SNP genotyping data was analyzed using the Genotyping module of the GenomeStudio software package (Illumina Inc., San Diego, CA, USA), as described previously [52]. Briefly, high-quality SNPs were selected from the list of all SNPs evaluated for genotyping using the following logical expression filter features in GenomeStudio (i) Call Freq: >0.50, (ii) Minor Allele Freq: >0.03, (iii) AA Freq: !=1, (iv) AB Freq: !=1, and (v) BB Freq: !=1. The parental profiles of SNPs were compared, followed by the selection of identical SNPs to remove monomorphic SNPs together. SNPs that showed segregation distortion were removed using a ‘two-step’ strategy: In the first step, SNPs with allele frequencies <0.4 and >0.6 were removed; In the second step, SNPs that deviated significantly from 1:1 ratio on the bases of χ^2^ values, were removed. Only high-quality polymorphic markers clearly segregating for a 1:1 ratio were used for genetic linkage mapping.

Initially, markers were assigned to linkage groups (LGs) using the minimum spanning tree algorithm implemented in the MSTMAP software (version 2.0) [98] (available freely at GitHub). Distance and objective functions, i.e., Kosambi [99] and maximum likelihood (ML) [100], respectively, were utilized to calculate the recombination frequencies and order of LGs. Double recombinants were corrected after rescoring, and individual LGs were refined at a threshold of LOD = 3.0 and recombination frequency = 0.35 using the MapDisto software (version 1.7.7.011) [101]. LGs were assigned to wheat chromosomes based on the wheat 90K consensus SNP map [54]. Different LGs generated from the same chromosome were merged to a single LG using gradually less stringent cut off values, followed by recalculating the map length using MapDisto.

The QTL Cartographer (version 1.6) software package [102,103] was used to map main effect QTL (M-QTL) using a composite interval mapping (CIM) method with a regression method, i.e., forwards and backwards cofactor (*p* = 0.05). QTL significance thresholds were determined by 1000 permutations at a significance level of *p* = 0.05 to declare putative QTL. QTL (2.5 < LOD < threshold) were also reported when detected in at least one environment or pooled data reaching the significance level following the method described by Yi et al. [65]. QTL intervals were calculated using the entire CIM interval above the threshold/selected LOD score. QTLNetwork (version 2.0) [104] was used for two-locus analyses to identify additive (A), epistatic and QTL × environment interaction (A*E) effects of QTL. A Circos diagram representing the AAC Innova x AAC Tenacious linkage and QTL map with epistatic interactions in a circular network plot was generated using the R package OmicCircos (version 1.14.0) software [105].

## 5. Conclusions

This study provides new insights into the genetic basis of the FHB resistance of wheat cv AAC Tenacious, which is the first Canadian spring wheat cv to obtain a FHB ‘resistant’ rating. In this study, we identified stable as well as environment-specific, pleotropic and epistatic FHB-resistance QTL. Notably, two important major, stable FHB-resistance QTL, mapped on wheat chromosome arms 2DS and 2DL, were derived from AAC Tenacious. While 2DS QTL is colocated with DTA and PHT QTL, 2DL is a solitary FHB responsive QTL. The latter is very important and may be useful to pyramid FHB resistance from sources such as Sumai 3 and others. The QTL identified in the present study will be valuable resources, not only for Canadian wheat breeders, but also for breeders working on similar wheat classes around the globe. However, environment-specific QTL should be subject to further investigation using a suitable approach such as meta-analysis to see if they possess any significant effect on FHB, for example FHB susceptibility.

## Figures and Tables

**Table 1:** Combined ANOVA table for disease incidence (DI), severity (DS), visual rating index (VRI = DI x DS) and deoxynivalenol content (DON) level of checks, parents and DH population AAC Innova x AAC Tenacious grown at Morden, Canada from 2015 to 2017.

Source	d.f.	DI	DS	VRI	DON
Environment (E)	1	20.25 **	175.80 **	967.00 *	8135.00 **
Block (within E)	1	9.91	0.40	172.00	94.00
Genotype (G)	236	31.19 **	20.60 **	2864.00 **	1094.00 **
(G х E)	236	3.22	2.70	237.00	130.00 **
Error	875	2.93	2.30	250.00	83.00
CV, %		24.00	31.00	40.00	41.00

CV: coefficient of variation; d.f.: degrees of freedom; Signif. codes: *p* ** ≤ 0.01, *p* * ≤ 0.05.

**Table 2:** Details of number of mapped molecular markers, map length and marker densities of individual linkage groups (LGs), seven homoeologous groups and three wheat genomes (A, B and D).

Genome	LG/ Chromosome	No. of Markers	No. of Linkage Bins	Map Length (cM)	Marker Density		Map Density	
					Markers/cM	Linkage Bins/cM	cM/ Marker	cM/Linkage Bin
**A**	1	737.00	124.00	187.48	3.93	0.66	0.25	1.51
	2	626.00	107.00	178.64	3.50	0.60	0.29	1.67
	3	430.00	87.00	237.23	1.81	0.37	0.55	2.73
	4	406.00	72.00	176.58	2.30	0.41	0.43	2.45
	5	760.00	116.00	232.93	3.26	0.50	0.31	2.01
	6	432.00	69.00	187.11	2.31	0.37	0.43	2.71
	7	721.00	138.00	297.25	2.43	0.46	0.41	2.15
**Total**		4112.00	713.00	1497.23	2.75	0.48	0.36	2.10
**%**		39.81	46.21	41.44				
**B**	1	1721.00	88.00	140.42	12.26	0.63	0.08	1.60
	2	817.00	109.00	202.65	4.03	0.54	0.25	1.86
	3.1	89.00	28.00	38.94	2.29	0.72	0.44	1.39
	3.2	385.00	68.00	113.39	3.40	0.60	0.29	1.67
	4	302.00	60.00	119.83	2.52	0.50	0.40	2.00
	5	576.00	103.00	264.67	2.18	0.39	0.46	2.57
	6	693.00	87.00	206.49	3.36	0.42	0.30	2.37
	7	494.00	95.00	194.88	2.53	0.49	0.39	2.05
**Total**		5077.00	638.00	1281.26	3.96	0.50	0.25	2.01
**%**		49.16	41.35	35.46				
**D**	1	107.00	33.00	79.61	1.34	0.41	0.74	2.41
	2	552.00	45.00	166.82	3.31	0.27	0.30	3.71
	3.1	25.00	6.00	18.14	1.38	0.33	0.73	3.02
	3.2	19.00	10.00	46.52	0.41	0.21	2.45	4.65
	3.3	87.00	7.00	26.64	3.27	0.26	0.31	3.81
	4	52.00	26.00	145.98	0.36	0.18	2.81	5.61
	5	156.00	30.00	181.74	0.86	0.17	1.16	6.06
	6	66.00	12.00	31.22	2.11	0.38	0.47	2.60
	7.1	45.00	9.00	23.12	1.95	0.39	0.51	2.57
	7.2	30.00	14.00	115.09	0.26	0.12	3.84	8.22
**Total**		1139.00	192.00	834.87	1.36	0.23	0.73	4.35
**%**		11.03	12.44	23.10				
**A+B+D**	1	2565.00	245.00	407.50	6.29	0.60	0.16	1.66
	2	1995.00	261.00	548.11	3.64	0.48	0.27	2.10
	3	1035.00	206.00	480.86	2.15	0.43	0.46	2.33
	4	760.00	158.00	442.39	1.72	0.36	0.58	2.80
	5	1492.00	249.00	679.34	2.20	0.37	0.46	2.73
	6	1191.00	168.00	424.82	2.80	0.40	0.36	2.53
	7	1290.00	256.00	630.34	2.05	0.41	0.49	2.46
**Total**		10328.00	1543.00	3613.36	2.86	0.43	0.35	2.34

**Table 3:** Details of quantitative trait loci identified for *Fusarium* head blight response variables/traits disease incidence (DI), severity (DS), visual rating index (VRI = DI x DS), deoxynivalenol content (DON), and days to anthesis (DTA) and plant height (PHT) on different wheat chromosomes in AAC Innova x AAC Tenacious doubled haploid population using pooled data of different environments.

Response Variable/ Trait	Chr. (arm)	QTL Name ^a^	QTL Position ^b^	QTL Interval	LOD	Absolute Additive Effect	*R* ^2^	Closest Marker				Individual Environment(s)	Favourable Donor Allele for Respective Trait
								Marker Name	Linkage Map Position	Physical Position (IWGSC RefSeq v2.0) ^c^ Start End			
DI	2B	*QFhi.lrdc-2B*	68.8	64.2–71.8	3.2	0.5	4.4	BS00012078_51	66.73	265366661	265366634	Mrd16, Mrd17	T
	2DS	QFhi.lrdc-2D*	40.9	40.2–63.7	15.5	1.0	25.0	Ppd-D1	40.76	36205433	36204772	Mrd15, Mrd16, Mrd17	T
	4A	*QFhi.lrdc-4A*	118.0	114.8–121.6	8.0	0.7	11.9	BobWhite_c10610_1096	117.82	688409042	688408942	Mrd16, Mrd17	I
	4D	*QFhi.lrdc-4D*	146.0	145.1–146.0	2.8	0.4	4.0	BobWhite_c28101_376	145.98	515801764	515801671	Mrd16, Mrd17	I
	5D	*QFhi.lrdc-5D*	0.1	0.0–0.6	2.6	0.4	3.9	RAC875_c92929_92	0.00	279491136	279491036	Mrd17	T
	7A	*QFhi.lrdc-7A*	206.4	205.9–206.8	2.6	0.4	2.1	IACX2471	205.99	673715328	673715447	--	T
DS	2DS	QFhs.lrdc-2D.1*	40.9	40.2–63.4	12.7	0.8	19.0	Ppd-D1	40.76	36205433	36204772	Mrd15, Mrd16, Mrd17	T
	2DL	QFhs.lrdc-2D.2*	115.6	105.2–126.4	5.4	0.5	15.2	BobWhite_c17782_194	115.38	555098707	555098805	Mrd15, Mrd16	T
	4A	*QFhs.lrdc-4A*	118.0	114.8–121.3	5.4	0.5	10.1	BobWhite_c10610_1096	117.82	688409042	688408942	Mrd15, Mrd16, Mrd17	I
	7BS	*QFhs.lrdc-7B*	32.3	24.3–35.5	3.6	0.4	6.3	wsnp_CAP7_c44_26549	32.15	17091249	17091049	Mrd16	I
VRI	2DS	QFhb.lrdc-2D.1*	40.9	40.2–63.4	12.4	9.4	18.0	Ppd-D1	40.76	36205433	36204772	Mrd15, Mrd16, Mrd17	T
	2DL	QFhb.lrdc-2D.2*	114.6	106.4–120.8	3.6	4.8	12.2	BobWhite_c17782_194	115.38	555098707	555098805	Mrd15	T
	4A	*QFhb.lrdc-4A*	118.0	114.7–121.3	7.1	6.6	11.5	BobWhite_c10610_1096	117.82	688409042	688408942	Mrd15, Mrd16, Mrd17	I
	5D	*QFhb.lrdc-5D*	0.2	0.0–0.4	2.5	3.4	3.0	RAC875_c92929_92	0.00	279491136	279491036	Mrd17	T
	7BS	*QFhb.lrdc-7B*	31.6	24.1–35.2	3.9	4.9	7.3	wsnp_CAP7_c44_26549	32.15	17091249	17091049	Mrd16	I
DON	2DS	QDon.lrdc-2D.1*	40.6	37.9–41.9	3.9	3.0	12.3	Ppd-D1	40.76	36205433	36204772	Mrd16, Mrd17	T
	2DL	QDon.lrdc-2D.2*	112.6	105. 1–126.6	14.4	6.0	34.5	BobWhite_c17782_194	115.38	555098707	555098805	Mrd15, Mrd16, Mrd17	T
	3B	QDon.lrdc-3B.1*	132.8	132.2–133.2	2.7	2.1	9.3	Kukri_c13345_481	132.75	753508932	753508832	Mrd15	T
	3B	*QDon.lrdc-3B.2*	147.3	144.5–150.3	2.7	2.3	4.0	Excalibur_rep_c97324_623	151.49	771943609	771943709	--	T
	4B	QDon.lrdc-4B*	49.5	46.4–52.4	6.6	3.6	8.0	Ex_c101685_705	49.48	31725418	31725518	Mrd17	T
	5A	*QDon.lrdc-5A.1*	164.8	156.4–169.0	3.1	2.3	3.0	IACX2540	164.62	621553818	621553699	Mrd16, Mrd17	T
	7BS	QDon.lrdc-7B*	52.9	52.2–57.7	3.4	2.7	5.0	Kukri_c19823_491	52.69	60918162	60918262	Mrd15	I
DTA	2B	*QDta.lrdc-2B*	65.9	64.9–66.7	2.6	0.6	3.0	BS00065276_51	66.14	57526894	57526994	Mrd17, Let18	I
	2DS	*QDta.lrdc-2D.1*	41.9	40.2–63.5	22.9	2.1	30.0	RAC875_c7319_195	41.83	36481727	36481627	Mrd17, Let17, Let18, Let19	I
	4A	*QDta.lrdc-4A.2*	120.0	117.4–123.8	6.8	1.1	7.0	Excalibur_c4325_1440	121.65	686650643	686650543	Mrd17, Let18, Let19	T
	7BS	*QDta.lrdc-7B*	27.3	18.1–31.9	6.6	1.1	8.0	IACX198	24.26	10548520	10548633	Mrd17, Let17, Let18, Let19	T
	7D	*QDta.lrdc-7D.1*	79.9	77.8–91.5	2.78	1.2	5.8	D_GCE8AKX02ILA1U_88	79.8186	56638676	56638464	Let17, Let18, Let19	T
	7D	*QDta.lrdc-7D.2*	117.7	113.0–118.0	3.4	0.8	3.5	wsnp_Ex_c10430_17064001	118.58	114113970	114114170	Mrd17	T
PHT	1B	*QPht.lrdc-1B.1*	36.0	33.0–38.5	7.1	2.4	6.0	IAAV4702	34.94	544295939	544296139	Mrd17, LetPGH17	I
	2DS	*QPht.lrdc-2D.1*	41.9	40.2–63.5	11.1	3.2	10.0	RAC875_c7319_195	41.83	36481727	36481627	Mrd17, Let17, Let18, Let19	I
	4A	*QPht.lrdc-4A.1*	22.6	17.8–26.8	2.6	1.5	2.0	BS00092244_51	26.38	11756178	11756078	Mrd17	T
	4B	*QPht.lrdc-4B*	49.6	46.0–52.6	42.0	7.3	53.0	Ex_c101685_705	49.48	31725418	31725518	Mrd17, Let17, LetPGH17, Let18, Let19	I
	5A	*QPht.lrdc-5A.2*	97.8	91.9–103.0	3.7	1.7	3.0	RAC875_c25072_389	97.75	524944276	524944376	Let17, Let18	T
	5D	*QPht.lrdc-5D*	26.5	24.7–42.5	2.9	1.7	3.0	wsnp_JD_c3690_4731341	26.59	401527745	401527907	--	T
	7D	*QPht.lrdc-7D.1*	79.9	67.9–95.2	5.0	2.0	4.0	D_GCE8AKX02ILA1U_88	79.82	56638676	56638464	Mrd17, Let17, Let18, Let19	T

Note: Chr.: chromosome; QTL: quantitative trait loci; LOD: logarithm of the odds score; *R*^2^: percent of the phenotypic variance; Mrd: Morden; Let: Lethbridge; PGH: green house; 17: 2017; 18: 2018; 19: 2019; ^a^ QTL were named according to all loci identified using pooled (average of all environments) as well as individual environment phenotypic data; ^b^ QTL positions were determined by the highest LOD peak positions (cM) on linkage groups; ^c^ Physical positions were determined by SNP probe sequence mapped to reference genome; -- QTL detected using pooled data only; I = AAC Innova; T = AAC Tenacious; * loci also appeared during epistasis QTL analysis.

**Table 4:** Details of epistasis quantitative trait loci (QTL) identified for disease incidence (DI), severity (DS), visual rating index (VRI = DI x DS), deoxynivalenol content (DON), and days to anthesis (DTA) and plant height (PHT) on different wheat chromosomes (chrs) in AAC Innova x AAC Tenacious doubled haploid population.

Trait	QTL_i ^a^	Chr	Interval_i ^b^	Position_i ^c^	Range_i ^d^	QTL_j ^a^	Chr	Interval_j^b^	Position_j ^c^	Range_j ^d^	AA ^e^	SE ^f^
DI	1–12	1A	BS00103478_51-BS00081682_51	17.7	12.7–23.2	2–86	1B	RAC875_c102886_73-Tdurum_contig9144_222	138.0	133.7–139.9	0.44	0.08 **
	5–82	2B	IAAV1743-Jagger_c3435_145	118.4	115.5–126.3	13–83	5A	RAC875_rep_c109969_119-RFL_contig316_572	151.3	148.0–161.4	−0.41	0.10 **
	6–11 ^#^	2D	Ppd-D1-wsnp_CAP12_c1503_764765	38.9	36–44.4	5–51 ^#^	2B	Tdurum_contig53156_111-Tdurum_contig1653_190	81.3	72.6–82.5	−0.38	0.08 **
	6–42	2D	RAC875_c5998_1056-D_F5MV3MU01EDEO3_100	145.5	142.6–155.6	4–6	2A	Excalibur_rep_c106338_424-RAC875_c54668_102	36.5	25.5–49.0	−0.51	0.09 **
DS	6–10 ^#^	2D	wsnp_CAP12_c812_428290-RAC875_c7319_195	38.1	34.5–42.4	6–22 ^#^	2D	BobWhite_c17782_194-TA002913-0806	115.4	106.2–120.4	−0.20	0.08 **
VRI	6–10 ^#^	2D	wsnp_CAP12_c812_428290-RAC875_c7319_195	38.1	36.1–39.4	6–22 ^#^	2D	BobWhite_c17782_194-TA002913-0806	112.4	108.2–119.4	−2.90	0.98 **
	10–41	4A	RAC875_c6075_214-BobWhite_c35402_66	84.8	84.2–87.8	14–7	5B	RAC875_c39204_91-TA015732-1144	20.6	12.6–27.6	6.40	0.89 **
DON	6–10 ^#^	2D	wsnp_CAP12_c812_428290-RAC875_c7319_195	38.1	28.5–48.4	11–17 ^#^	4B	Ex_c101685_705-Tdurum_contig42229_113	49.5	47.7–50.0	2.02	0.41 **
	6–21 ^#^	2D	wsnp_ku_c8712_14751858- BobWhite_c17782_194	107.2	104.2–110.2	8–69 ^#^	3B	Kukri_c13345_481-wsnp_Ex_c19778_28779907	132.7	130.1–135.3	1.52	0.57 **
	8–12	3B	RAC875_rep_C107068_182-RAC875_rep_C69171_241	6.7	0.0–18.4	17–12	6B	IAAV5385-Tdurum_contig61178_618	28.5	25.8–34.5	1.92	0.53 **
	8–69 ^#^	3B	Kukri_c13345_481-wsnp_Ex_c19778_28779907	132.7	130.1–135.3	20–16 ^#^	7B	Kukri_c19823_491-CAP11_rep_c8279_82	53.7	52.1–58.3	−1.65	0.54 **
	13–9	5A	Ex_C95453_1499-Excalibur_c13536_202	20.3	16.3–33.3	13–98 ^#^	5A	Kukri_c108256_381-BS00090847_51	195.7	184.7–196.9	−1.93	0.43 **
	13–52	5A	wsnp_Ku_c15816_24541162-GENE-3314_78	88.4	87.9–91.1	21–12	7D	D_GCE8AKX02ILA1U_88-D_CONTIG12156_209	103.8	94.8–111.9	−3.62	0.46 **
DTA	6–10 ^#^	2D	wsnp_CAP12_c812_428290-RAC875_c7319_195	38.1	36.1–40.4	19–123	7A	Wsnp_Ku_c8437_14341371-Excalibur_c3188_1352	234.8	228.3–238.5	0.55	0.20 **
PHT	6–12 ^#^	2D	wsnp_CAP12_c1503_764765-wsnp_Ku_c12022_19520410	39.4	36.1–45.4	7–35 ^#^	3A	Excalibur_c19671_139-wsnp_Ex_c9458_15679797	88.9	85.0–92.2	−0.67	0.31 *
	11–17 ^#^	4B	Ex_c101685_705-Tdurum_contig42229_113	49.5	48.7–50.0	21–12 ^#^	7D	D_GCE8AKX02ILA1U_88-D_CONTIG12156_209	79.8	74.0–85.8	−1.08	0.31 **

Note: ^a^ QTL_i and QTL_j: The two QTL involved in an epistatic interaction. QTL are named with the relevant chromosome and the marker intervals. The first (before ‘–’) and second (after ‘–’) part of QTL name represents chr number (1 to 21) and marker interval, respectively on relevant chromosome; ^b^ Interval_i and interval_j: The flanking markers of QTL_i and QTL_j, respectively; ^c^Position_i and position_j: The distance between QTL_i/QTL_j and the first marker of the relevant chromosome; ^d^ Range_i and range_j: The position support intervals of QTL_i and QTL_j, respectively; ^e^ AA: The estimated additive x additive (epistatic) effect. A positive epistasis value means that the phenotype is higher than expected, while a negative epistasis value means that the phenotype is lower than expected.; ^f^ SE: The standard error of estimated or predicted QTL effect; significance codes: *p* ** ≤0.01, *p* * ≤0.05; ^#^ loci also appeared during main effect QTL analysis.

## Supplementary Materials

Supplementary materials can be found at https://www.mdpi.com/1422-0067/21/12/4497/s1.

## Abbreviations

AA	Additive × additive (epistatic) effect
AE	Additive effect
A*E	QTL × environment interaction
ANOVA	Analysis of variance
Chr	Chromosome
CIM	Composite interval mapping
cM	centi-Morgan
cm	Centimetre
CPS	Canada Prairie Spring wheat class
CWSP	Canada Western Special Purpose wheat class
cv	Cultivar
CV	Coefficient of variation
df	Degrees of freedom
DH	Doubled haploid
DI	Disease incidence
DON	Deoxynivalenol
DS	Disease severity
DTA	Days to anthesis
E	Environment
FDK	*Fusarium* damaged kernels
Fg	Fusarium graminearum (Schwabe)
FHB	*Fusarium* head blight
G	Genotype
GA	Gibberellic acid
GDD	Growing degree days
His	Histidine-rich calcium-binding protein
I	Intermediate
JD	Julian calendar days
LG	Linkage group
LOD	Logarithm of the odds
MR	Moderately resistant
MS	Moderately susceptible
PFT	Pore-forming toxin-like protein
PHT	Plant height
QTL	Quantitative trait loci
R	Resistant
R^2^	Phenotypic variation
Rht	Reduced height
S	Susceptible
SNP	Single nucleotide polymorphism
SSR	Simple sequence repeat
VRI	Visual rating index
