# Supplementary material for: Mapping of Major Fusarium Head Blight Resistance from Canadian Wheat cv. AAC Tenacious
Source: Int J Mol Sci. 2020 Jun 24;21(12):4497. doi: 10.3390/ijms21124497 (PMC7350018; doi:10.3390/ijms21124497)
Supplement: Supplementary file 1 [file ijms-21-04497-s001.zip › Supplementary Table S1.docx]

**Supplementary Table S1:** Summary of *Fusarium* head blight (FHB) response traits disease incidence (DI; rated on a scale of 0-10), severity (DS; rated on a scale of 0-10), visual rating index (VRI: DI × DS) and deoxynivalenol content (DON; ppm) of AAC Innova and AAC Tenacious, their doubled haploid progenies and checks evaluated at Morden, Canada during 2015, 2016 and 2017.

| **Trait and Env** | **Parental lines** | | **Population** | | | **Checks** | | | | | | | | | | **%CV**  **(LSD)** |
| --- | --- | --- | --- | --- | --- | --- | --- | --- | --- | --- | --- | --- | --- | --- | --- | --- |
|  | **AAC Innova** | **AAC Tenacious** | **Min** | **Max** | **Mean** | **AC Sadash** | **AAC Foray** | **AC Carberry** | **AAC Indus** | **AAC Penhold** | **CDC Teal** | **AC Morse** | **AC Cora** | **5602 HR** | **FHB 37** |  |
| **FHB DI (Type-I resistance)** | | | | | | | | | | | | | | | | |
| **2015** | 7.1 | 2.5 | 2.0 | 9.5 | 6.3 | 9.1 | 8.0 | 8.0 | 7.5 | 9.0 | 9.1 | 8.4 | 6.2 | 7.2 | 5.4 | 24.0 |
| **2016** | 7.9 | 4.4 | 0.2 | 10.0 | 7.2 | 9.8 | 9.5 | 10.0 | 7.3 | 9.8 | 10.0 | 10.0 | 8.9 | 9.7 | 8.6 | (0.25) |
| **2017** | 8.2 | 2.2 | 0.2 | 10.0 | 6.7 | 10.0 | 9.8 | 9.9 | 8.5 | 9.8 | 9.6 | 9.9 | 7.8 | 9.3 | 9.0 |  |
| **M** | 7.7 | 3.0 | 0.8 | 9.8 | 6.7 | 9.6 | 9.1 | 9.3 | 7.8 | 9.5 | 9.6 | 9.4 | 7.6 | 8.7 | 7.7 |  |
| **FHB DS (Type-II resistance)** | | | | | | | | | | | | | | | | |
| **2015** | 6.2 | 3.2 | 2.0 | 9.0 | 5.5 | 8.3 | 5.9 | 7.5 | 6.0 | 5.0 | 7.8 | 7.8 | 5.4 | 6.4 | 2.0 | 31.0 |
| **2016** | 5.6 | 2.4 | 0.5 | 9.7 | 4.9 | 8.0 | 8.5 | 5.5 | 6.0 | 6.0 | 8.1 | 7.9 | 5.4 | 6.0 | 5.1 | (0.21) |
| **2017** | 6.4 | 1.7 | 0.2 | 9.5 | 4.4 | 8.0 | 6.5 | 5.1 | 6.3 | 4.3 | 7.8 | 6.8 | 5.2 | 5.7 | 4.0 |  |
| **M** | 6.1 | 2.4 | 0.9 | 9.4 | 4.9 | 8.1 | 7.0 | 6.0 | 6.1 | 5.1 | 7.9 | 7.5 | 5.3 | 6.0 | 3.7 |  |
| **FHB VRI** | | | | | | | | | | | | | | | | |
| **2015** | 43.5 | 8.5 | 6.0 | 76.5 | 36.6 | 75.4 | 46.9 | 60.0 | 45.0 | 45.0 | 70.3 | 65.5 | 34.2 | 46.2 | 11.4 | 40.0 |
| **2016** | 46.4 | 13.6 | 0.2 | 97.5 | 41.7 | 78.0 | 81.0 | 55.0 | 45.3 | 58.5 | 80.2 | 79.0 | 48.3 | 58.0 | 46.7 | (2.25) |
| **2017** | 52.9 | 4.3 | 0.1 | 95.0 | 35.0 | 80.0 | 63.8 | 50.8 | 53.3 | 41.4 | 74.0 | 67.1 | 40.6 | 52.7 | 35.8 |  |
| **M** | 47.6 | 8.8 | 2.1 | 89.7 | 37.8 | 77.8 | 63.9 | 55.3 | 47.9 | 48.3 | 74.8 | 70.5 | 41.0 | 52.3 | 31.3 |  |
| **DON (Type-III resistance)** | | | | | | | | | | | | | | | | |
| **2015** | 25.0 | 1.4 | 0.6 | 95.8 | 15.5 | 40.1 | 43.2 | 16.2 | 36.1 | - | 51.3 | 63.6 | 17.4 | 28.0 | 6.8 | 41.0 |
| **2016** | 41.6 | 6.8 | 3.6 | 82.4 | 20.7 | 65.6 | 40.7 | 19.6 | 36.7 | 23.5 | 43.0 | 60.0 | 17.8 | 29.6 | 15.1 | (1.14) |
| **2017** | 43.6 | 4.1 | 1.5 | 70.1 | 22.8 | 68.2 | 52.8 | 39.2 | 56.4 | 50.2 | 33.7 | 61.4 | 14.6 | 28.7 | 23.9 |  |
| **M** | 36.7 | 4.1 | 1.9 | 82.8 | 19.7 | 58.0 | 45.6 | 25.0 | 43.1 | 36.9 | 42.7 | 61.7 | 16.6 | 28.8 | 15.3 |  |

Note: Env: Environment; M: mean of locations; - : respective check was not used for the given environment.
