# Supplementary material for: Mapping of Major Fusarium Head Blight Resistance from Canadian Wheat cv. AAC Tenacious
Source: Int J Mol Sci. 2020 Jun 24;21(12):4497. doi: 10.3390/ijms21124497 (PMC7350018; doi:10.3390/ijms21124497)
Supplement: Supplementary file 1 [file ijms-21-04497-s001.zip › Supplementary Table S2.docx]

**Supplementary Table S2:** ANOVA table for days to anthesis (DTA) of 196 lines belonging to check cultivars, parents and doubled haploid population AAC Innova x AAC Tenacious grown at Lethbridge and Morden, Canada during 2017 to 2019.

| **Source** | **df** | **Mean Sq** | **F value** | **Pr(>F)** |
| --- | --- | --- | --- | --- |
| Environment (E) | 3 | 38286 | 8859.8781 | < 2.2e-16 ** |
| Treatment (T) | 195 | 61 | 14.2248 | 4.718e-16 ** |
| (E х T) | 584 | 7 | 1.7247 | 0.01737 * |
| Error | 40 | 4 |  |  |

Note: df: degrees of freedom; Signif. codes: ‘**’ 0.01 ‘*’ 0.05
