# Supplementary material for: Mapping of Major Fusarium Head Blight Resistance from Canadian Wheat cv. AAC Tenacious
Source: Int J Mol Sci. 2020 Jun 24;21(12):4497. doi: 10.3390/ijms21124497 (PMC7350018; doi:10.3390/ijms21124497)
Supplement: Supplementary file 1 [file ijms-21-04497-s001.zip › Supplementary Table S3.docx]

**Supplementary Table S3:** ANOVA table for plant height (PHT) of 196 lines belonging to check cultivars, parents and doubled haploid population AAC Innova x AAC Tenacious grown at Lethbridge and Morden, Canada during 2017 to 2019.

| **Source** | **df** | **Mean Sq** | **F value** | **Pr(>F)** |
| --- | --- | --- | --- | --- |
| Environment (E) | 4 | 19856.5 | 787.1876 | <2e-16 ** |
| Treatment (T) | 195 | 501.3 | 19.8726 | <2e-16 ** |
| (E х T) | 779 | 35.7 | 1.4134 | 0.0627 |
| Error | 50 | 25.2 |  |  |

Note: df: degrees of freedom; Signif. codes: ‘**’ 0.01 ‘*’ 0.05
