# Supplementary material for: Mapping of Major Fusarium Head Blight Resistance from Canadian Wheat cv. AAC Tenacious
Source: Int J Mol Sci. 2020 Jun 24;21(12):4497. doi: 10.3390/ijms21124497 (PMC7350018; doi:10.3390/ijms21124497)
Supplement: Supplementary file 1 [file ijms-21-04497-s001.zip › Supplementary Table S4.docx]

**Supplementary Table S4:** Summary of days to anthesis (DTA) and plant height (PHT) of checks, parents and doubled haploid population AAC Innova x AAC Tenacious grown at Lethbridge and Morden in Canada during 2017, 2018 and 2019.

| **Trait and Env** | **Parental lines** | | **Population** | | | **Checks** | | | | | | | | | | **%CV (LSD)** |
| --- | --- | --- | --- | --- | --- | --- | --- | --- | --- | --- | --- | --- | --- | --- | --- | --- |
|  | **AAC Innova** | **AAC Tenacious** | **Min** | **Max** | **Mean** | **AC Sadash** | **AAC Foray** | **AC Carberry** | **AAC Indus** | **AAC Penhold** | **CDC Teal** | **AC Morse** | **AC Cora** | **5602 HR** | **FHB 37** |  |
| **Days to anthesis** | | | | | | | | |  |  |  |  |  |  |  |  |
| **2017MF** | 68.8 | 66.5 | 59.5 | 79.5 | 67.7 | 65.5 | 66.5 | 60.3 | 64.5 | 64.5 | 63.9 | 66.4 | 65.1 | 64.8 | 65.9 | 2.7 |
| **2017LG** | 90.6 | 87.2 | 73.0 | 108.0 | 86.3 | 76.0 | 77.0 | 72.0 | 90.0 | 73.0 | - | - | - | - | - | (0.41) |
| **2018LF** | 58.3 | 55.0 | 47.0 | 69.0 | 55.9 | 52.0 | 50.0 | 48.5 | 55.0 | 50.0 | - | - | - | - | - |  |
| **2019LF** | 62.8 | 58.5 | 50.0 | 73.0 | 59.7 | 55.0 | 58.0 | 52.0 | 57 | 54.0 | - | - | - | - | - |  |
| **M** | 70.1 | 66.8 | 57.4 | 82.4 | 67.4 | 62.1 | 62.9 | 58.2 | 66.6 | 60.4 | - | - | - | - | - |  |
| **Plant height (cm)** | | | | | | | | | | | | | | | |  |
| **2017MF** | 89.0 | 107.3 | 69.0 | 128.0 | 97.3 | 86.0 | 90.0 | 84.0 | 95.0 | 77.5 | 97.8 | 96.0 | 105.4 | 97.4 | 85.5 | 4.6 |
| **2017LG** | 95.7 | 111.9 | 70.6 | 131.5 | 101.9 | 105.2 | 112.2 | 90.9 | 93.5 | 83.5 | - | - | - | - | - | (0.99) |
| **2017LF** | 74.2 | 95.7 | 57.3 | 108.0 | 83.3 | 77.0 | 77.0 | 78.2 | 70.3 | 69.7 | - | - | - | - | - |  |
| **2018LF** | 101.2 | 112.8 | 79.6 | 136.0 | 109.6 | 98.3 | 98.0 | 87.1 | 100.6 | 78.3 | - | - | - | - | - |  |
| **2019LF** | 85.7 | 98.7 | 68.7 | 121.7 | 91.7 | 85.0 | 80.66666667 | 78.0 | 84.7 | 70.3 | - | - | - | - | - |  |
| **M** | 89.2 | 105.3 | 69.1 | 125.8 | 98.0 | 86.5 | 97.06 | 74.44 | 92.5 | 79.7 | - | - | - | - | - |  |

Note: Env: Environment; MF: Morden field; LG: Lethbridge green house; LF: Lethbridge field; M: mean of locations; - : data not collected/utilized.
